# Current and Desired Quality of Life in People with Parkinson’s Disease: The Calman Gap Increases with Depression

**DOI:** 10.3390/jcm9051496

**Published:** 2020-05-15

**Authors:** Tino Prell, Ulrike Teschner, Otto W. Witte, Albrecht Kunze

**Affiliations:** 1 Department of Neurology, Jena University Hospital, 07747 Jena, Germany; Ulrike.Teschner@med.uni-jena.de (U.T.); otto.witte@med.uni-jena.de (O.W.W.); albrecht.kunze@med.uni-jena.de (A.K.); 2 Center for Healthy Ageing, Jena University Hospital, 07747 Jena, Germany

**Keywords:** quality of life, Calman gap, depression, pain, physical activity

## Abstract

Hopes and expectations often differ from current experiences. This so-called Calman gap influences quality of life (QoL). We investigated this gap in 77 elderly patients with Parkinson’s disease (PD), 25 patients with epilepsy, and 39 age-matched healthy older adults using a novel QoL questionnaire, where current and desired states were marked on a visual analogue scale. We studied the relationships between (1) epidemiological factors, (2) current and desired QoL, as well as the difference between the latter two. Current QoL was determined by depression, education level, living situation, and condition (PD, epilepsy, control). In contrast, desired QoL was essentially determined by the presence of a disease (condition), education level, and age, but not by depression. In particular, the presence of PD, lower education level, and higher age was correlated with lower expectations. In patients with PD, the gap between the current and desired QoL was largest for pain and physical functions. Accordingly, the significant effects of depression were observed only for mean current QoL, but not for desired QoL. Therefore, depression mainly influences current but not desired QoL in patients with PD. Depressed patients with PD had significantly worse QoL than PD patients without depression, although they both had almost the same desired QoL and hence, depressed PD patients had a larger Calman gap between current and desired QoL.

## 1. Introduction

Every person has different desires, expectations, and dreams, which are usually different from their current state of living. This difference or gap between the hopes/expectations and actual experiences of an individual (the so-called Calman gap) influences their quality of life (QoL) and satisfaction [1]. If the individual’s current condition worsens in the context of a disease (e.g., impairment of mobility in Parkinson’s disease [PD]), then the gap between the current and desired state may increase, thereby lowering the satisfaction of the individual. On the other hand, individuals can adapt their expectations and desires in certain domains and thus maintain a consistently good QoL despite the deterioration of their physical function (response shift).

Numerous predictors of health-related QoL have been reported for PD [2,3,4,5,6,7], but the Calman gap has not yet been studied. In addition, existing measures of QoL do not account for health expectations; expectations and experiences are not measured against specific levels. Patients have different understandings of their illness and other reference points, which can also change over time. This means that an individual with low expectations might not evaluate the experience of poor health as affecting their QoL. In contrast, in an individual with high expectations about his or her health, a minor illness might have a significant effect on their perceived QoL [8]. Examining the Calman gap would therefore greatly enhance our understanding of QoL by better understanding how patients with PD deal with the progressive loss of function.

In routine clinical practice, every physician encounters PD patients who suffer from unrealistic expectations regarding their physical functioning. For example, some patients who ordinarily demand too much from their own bodies cannot accept the fact that their motor skills are no longer as efficient as they were before the onset of PD. This observation was the starting point for our study. We developed a simple questionnaire in which patients indicated their current and desired states of living using a visual analogue scale (VAS). The domains surveyed included physical functions, occupational and financial aspects, and lifestyle topics. The questionnaires were filled out anonymously by patients attending an information session for PD patients. Individuals without motor impairments were recruited as controls: age-matched healthy controls and younger patients with epilepsy since epilepsy is another chronic disease associated with relevant restrictions (e.g., in the occupational or lifestyle domain). In addition to collecting general epidemiological data, we administered self-report questionnaires to determine whether depression was an important predictor of QoL.

This exploratory study aimed to answer the following main questions: (1) Do patients with PD have exaggerated expectations regarding their QoL? (2) Is there a subgroup of patients with PD that have a larger Calman gap?

## 2. Methods

### 2.1. Subjects and Assessments

Data were collected during two particular events for patients with PD and epilepsy in 2019. The events are held yearly at the Jena University Hospital, Jena, Germany and included lectures by neurologists on disease-related topics. The main participants were patients from the area of Thuringia, who were informed about the event in advance via flyers. The flyers were sent to neurology clinics and practices in Thuringia, as well as to self-support groups. For this study, 80 questionnaires were distributed for PD and 40 for epilepsy. The patients were asked to fill in the questionnaires anonymously at the beginning of the event and to put them into a ballot box after the event, so that no individual who filled out a questionnaire could be identified. Healthy control subjects were recruited via senior sports groups and the local hiking club (40 questionnaires were distributed in those settings).

The study was approved by the local ethics committee of the Jena University Hospital (4572-10/15). Due to the anonymous nature of the survey, no written informed consent was obtained. However, all subjects were informed in writing about the purpose of the study and how the data would be analyzed. In addition to the QoL questionnaire, the following data were also requested: age, gender, marital status (single; married; partnership; and divorced, widowed, or separated), living situation (alone; with partner/family; with others), education level (low [no school or German Hauptschule]; middle [high school or less]; high [university degree or German Abitur]), and occupation (employed full time; employed part-time; unemployed; retired). Depending on the patient event, we asked one of the following groups of questions: (1) Do you suffer from PD? If yes, when did you experienced the first symptoms related to PD? (2) Do you suffer from epilepsy? If yes, how many years have you had epilepsy? How often do you have epileptic seizures on average [none; every few years; every few months; weekly]? When was the last time you had an epileptic seizure [days ago, weeks ago, months ago, years ago]?

In addition, all the participants were requested to fill out the Patient Health Questionnaire-9 (PHQ-9). This is a self-administered instrument for screening, diagnosing, monitoring, and measuring the severity of depression. It includes nine items from the components of depression listed in the *Diagnostic and Statistical Manual of Mental Disorders, Fourth Edition*, each of which is scored on a scale of 0 (not at all) to 3 (every day). Total scores of 10, 15, and 20 represent cut-off points for mild, moderate, and severe depression, respectively [9].

### 2.2. Development of the Quality of Life Questionnaire

The 15 questions of the questionnaire were derived from existing and validated QoL and health-related QoL questionnaires: (1) Fragebogen zur Lebenszufriedenheit (Life Satisfaction Questionnaire) [10]; (2) Befragung “Generation 50 plus: Lebensqualität und Zukunftsplanung in Düsseldorf” (Generation 50 Plus: QoL and Future Planning in Düsseldorf) [11]; and (3) the Short Form (36) Health Survey [12]. A translation of the German version of the questionnaire is presented as a supplement in Table S1. The questionnaire covers the three dimensions of physical function, work/finance, and lifestyle.

**Figure 1:**
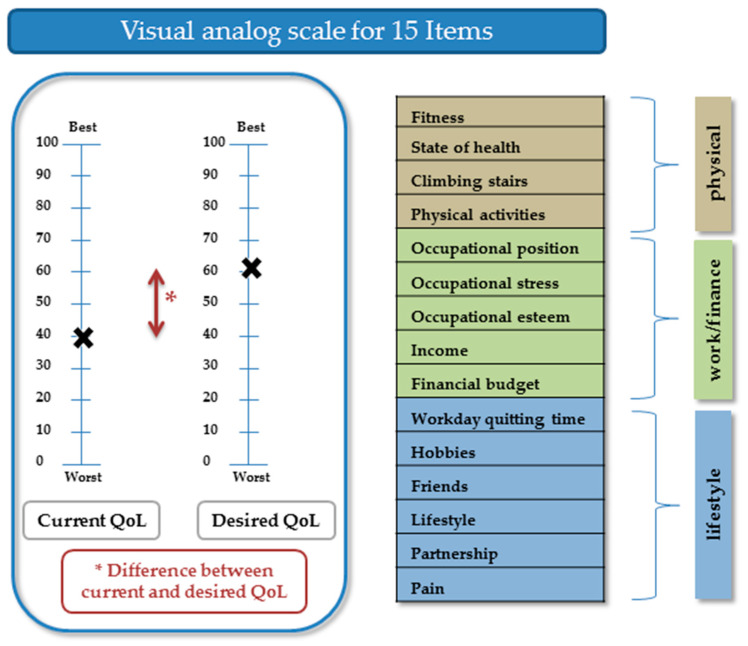
Quality of Life questionnaire. For the fifteen quality of life (QoL) questions, participants marked two visual analogue scales: one for current QoL and the other for desired QoL. A score of 100 indicated the best quality of life, while a score of 0 indicated the worst.

On each page of the questionnaire, two VASs were displayed: one representing current QoL and the other representing desired QoL. A VAS score of 100 indicated the best condition and 0 the worst condition (for pain, we used an inverse rating). The values entered by the participants along the VAS were recorded in steps of 5. For every item, current QoL (VAS value), desired QoL (VAS value), and the difference between the current and desired QoL were calculated (Figure 1). The Cronbach’s alpha level for current QoL was 0.87, for desired QoL 0.89, and for the difference between current and desired QoL 0.85.

### 2.3. Statistical Analysis

The SPSS statistical package (version 25.0; IBM Corporation, Armonk, NY, USA) was used for all statistical analyses. In the first step, we described the cohort using descriptive statistics. Correlation analyses were done using Pearson’s correlation for normally distributed data and Spearman’s correlation for skewed data. Comparisons of clinical variables between the two groups were performed with the *t* test for normally distributed data and the Mann-Whitney *U* test for skewed data. For comparisons involving more than two groups, an analysis of variance for normally distributed data and the Kruskal-Wallis test for skewed data were performed, along with the Dunn-Bonferroni test as a post hoc test. The chi-square or Fisher’s test was used to compare the categorical variables.

Several linear regressions were performed to explore the association between sociodemographic parameters and (1) current QoL, (2) desired QoL, and (3) the difference between the current and desired QoL (Akaike’s information criterion with stepwise forward selection). For the models, the following independent variables were entered into the regressions: age, gender, condition (PD, epilepsy, control), education level, marital status, living situation, employment status, and PHQ-9 score. The condition was entered in every model because the PHQ-9 differed between the three groups. The models were performed for the entire cohort, as well as for patients with PD only.

A multivariate analysis of variance (MANOVA) was used to study the effect of depression (PHQ-9) on the three QoL measures (current QoL; desired QoL; difference between current and desired QoL). A multivariate analysis of covariance (MANCOVA) was used to adjust these findings for age, gender, and condition. The MANOVA and MANCOVA were performed for the entire cohort, as well as for patients with PD only.

For all analyses, the significance level was set at p < 0.05. Values are given as means and standard deviations (SDs) or as medians and interquartile ranges (IQRs). Categorical variables are presented as numbers or percentages. Data from this study are available upon request to qualified investigators.

## 3. Results

### 3.1. Descriptive Statistics

The descriptive statistics of the demographic characteristics are shown in Table 1. The 77 patients with PD (mean age = 68.3 years, SD = 8.9 years; 95% CI, 66.3–70.3) and the 39 controls (mean age = 65.2 years, SD = 10.2 years; 95% CI, 61.9–68.5) were older than the 25 patients with epilepsy (mean age = 50.9 years, SD = 13.7 years; 95% CI, 44.8–57.0). Most of the patients with PD were married, were retired, lived with a partner, and had a medium or high education level. The mean disease duration in PD was 8.8 years (SD = 7.4) and in patients with epilepsy 25.7 years (SD = 17.1). Patients with epilepsy reported to have epileptic seizures on average none (*n* = 5, 21.7%), every few years (5, 21.7%), every few months (7, 30.4%), and weekly (6, 26.1%). The last epileptic seizure was days ago (*n* = 6, 24.0%), weeks ago (3, 12.0%), months ago (7, 28.0%), and years ago (9, 36.0%).

### 3.2. Evaluation of Current Quality of Life

**Figure 2:**
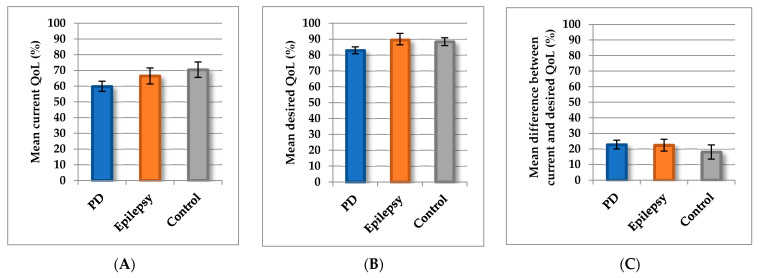
Quality of life in patients with Parkinson’s disease (PD), patients with epilepsy, and controls. (**A**) Mean current quality of life (QoL) according to patients with Parkinson’s disease (PD), patients with epilepsy, and controls (with 95%CI). (**B**) Mean desired QoL according to patients with PD, patients with epilepsy, and controls (with 95%CI). (**C**) Mean difference between current and desired QoL according to patients with PD, patients with epilepsy, and controls (with 95%CI).

We then explored the relationships between epidemiological and clinical factors and current QoL, desired QoL, and the difference between current and desired QoL. Current QoL was poorer in patients with PD (mean = 59.9, SD = 14.1; 95% CI, 56.7–63.1) than in controls (mean = 70.5, SD = 15.2; 95% CI, 65.6–75.4). Current QoL of the epilepsy patients was intermediate between those of patients with PD and controls (mean = 66.6, SD = 12.3; 95% CI, 61.6–71.8; Figure 2A). The current QoL of male (mean = 64.6, SD = 14.6; 95% CI, 60.9–68.3) and female (mean = 63.6, SD = 15.0; 95% CI, 60.2–66.9) participants did not differ (*p* = 0.69). The distinct items for the entire cohort are displayed in Supplement Figure S1A. Poorer scores for fitness, state of health, climbing stairs, physical activities, and lifestyle indicated poorer QoL in patients with PD than in patients with epilepsy or controls (Supplement Table S2).

In the linear regression for the entire cohort, the mean current QoL was predicted by the PHQ-9 sum score, education level, living situation, and condition (PD, epilepsy, control) (adjusted *R*^2^ = 0.39, *p* < 0.001) (Table 2). Also, in the cohort of PD patients, the mean current QoL was predicted by the PHQ-9 sum score, education level, and living situation (adjusted *R*^2^ = 0.27, *p* < 0.001) (Table 2).

### 3.3. Desired Quality of Life

Desired QoL was lower in patients with PD (mean = 82.8, SD = 9.5; 95% CI, 80.6–85.0) than in controls (mean = 88.4, SD = 7.6; 95% CI, 85.9–90.9; *p* = 0.002) and in patients with epilepsy (mean = 89.5, SD = 8.7; 95% CI, 85.4–92.6; *p* = 0.01) (Figure 2B). Patients with PD expected less QoL in all domains, although differences between those patients and the controls were significant only in the physical domains (climbing stairs *p* = 0.009, physical activities *p* = 0.007). In comparison with the younger patients with epilepsy, the PD patients (and controls) also desired a lower QoL state in terms of lifestyle, workday quitting time, and hobbies (detailed items in Supplement Table S2 and Figure S1B).

In the linear regression for the entire cohort, condition (PD, epilepsy, control), education level, and age were found to be significant predictors of desired QoL (adjusted *R*^2^ = 0.15, *p* < 0.001) (Table 3). In the cohort of PD patients, the desired current QoL was predicted by education level, age, and marital status (adjusted *R*^2^ = 0.14, *p* < 0.001) (Table 3). Of note, the group of PD patients included less people that were divorced/widowed/separated than the patients with epilepsy. This might contribute to the different predictors between the entire cohort and the PD patients.

### 3.4. Differences between Current and Desired Quality of Life

The scores for difference between current and desired QoL did not differ significantly among patients with PD (mean = 22.8, SD = 12.2; 95% CI, 20.0–25.6), controls (mean = 18.1, SD = 14.1; 95% CI, 13.5–22.6), and patients with epilepsy (mean = 22.4, SD = 9.3; 95% CI, 18.6–26.3; *p* = 0.14; Figure 2C). However, in contrast to controls and patients with epilepsy, the patients with PD showed greater differences between current and desired QoL in the physical domains (fitness, health state, climbing stairs, and physical activities) and finances (Supplement Table S2). In patients with PD, the largest difference between current and desired QoL state was reported for pain and for items of the physical domains (Supplement Figure S2). In contrast to controls, none of the patients with PD or epilepsy reported that they are fully satisfied with their QoL (for full satisfaction, the difference between current and desired QoL would be zero).

In the linear regression for the entire cohort, PHQ-9 was the strongest predictor of the difference between current and desired QoL state, followed by age and gender (adjusted *R*^2^ = 0.36, *p* < 0.001) (Table 4). In the cohort of PD patients, the difference between current and desired QoL was predicted by the PHQ-9 sum score, gender, and marital status (adjusted *R*^2^ = 0.24, *p* < 0.001) (Table 4).

All values for current, desired, and difference between current and desired QoL state are given in the Supplement Table S2.

### 3.5. The effect of Depression

**Figure 3:**
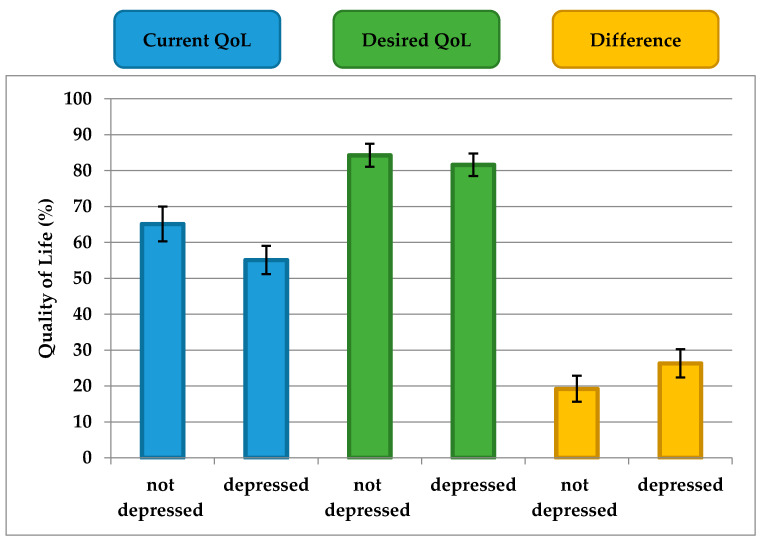
Depression and quality of life in Parkinons’s disease. Mean current quality of life (QoL), desired QoL, and difference between current and desired QoL in patients with Parkinson’s disease (mean and 95%CI).

Since previous analyses have shown that depression is a relevant cofactor, we included the following exploratory analyses. The mean PHQ-9 score was highest in patients with PD (mean = 9.6, SD = 4.4; 95% CI, 8.6–10.7), followed by patients with epilepsy (mean = 7.2, SD = 5.1; 95% CI, 5.1–9.3) and controls (mean = 5.7, SD = 4.9; 95% CI, 4.0–7.3). According to the PHQ-9, 16 participants of the entire cohort were moderately or severely depressed (scores for 6 subjects were missing; Table 1). To analyze these findings, the scores of the participants with mild, moderate, and severe depression (PHQ-9 ≥ 10) were grouped together (termed “depressed”) and the scores of the subjects with no depression (PHQ < 10) were grouped separately (termed “not depressed”). Current QoL and the difference between current and desired QoL differed between PD patients with and without depression (*p* = 0.002, *p* = 0.009, respectively) (Figure 3). However, the desired QoL did not significantly differ between PD patients with and without depression (*p* = 0.24) (Figure 3).

The MANOVA revealed a significant multivariate main effect of PHQ-9 (Wilks’s λ = 0.63, *p* < 0.001, η*_p_*^2^ = 0.37) on mean current QoL, desired QoL, and difference between current and desired QoL. The strongest univariate main effects of PHQ-9 were found for mean current QoL (*p* < 0.001, η*_p_*^2^ = 0.34) and for the difference between current and desired QoL (*p* < 0.001, η*_p_*^2^ = 0.31), followed by mean desired QoL (*p* = 0.038, η*_p_*^2^ = 0.032).

A MANCOVA was conducted to examine whether other medical covariates (age, gender, condition) could account for these findings. In this analysis, age (Wilks’s λ = 0.91, *p* = 0.008, partial η^2^ = 0.09), but not gender (*p* = 0.29) and condition (*p* = 0.70) was significant in the model. In addition, the results did not significantly change after we controlled for these variables, inasmuch as there remained a significant main effect of PHQ-9 score on mean current QoL (*p* < 0.001, η*_p_*^2^ = 0.30) and the difference between current and desired QoL (*p* < 0.001, η*_p_*^2^ = 0.32), but not on mean desired QoL (*p* = 0.25). Age had a weak effect on desired QoL (*p* = 0.024, η*_p_*^2^ = 0.04) and the difference between current and desired QoL (*p* = 0.005, η*_p_*^2^ = 0.06), but not on current QoL (*p* = 0.41).

As expected, there was a significant interaction between condition and PHQ-9 (Wilks’s λ = 0.67, *p* < 0.001, partial η^2^ = 0.13) in the analysis of the entire cohort. Performing the same analysis in the patients with PD only showed similar results: There was a significant multivariate main effect of PHQ-9 (Wilks’s λ = 0.72, *p* < 0.001, η*_p_*^2^ = 0.28) on mean current QoL, desired QoL, and difference between current and desired QoL. Strongest univariate main effects of PHQ-9 were found for mean current QoL (*p* < 0.001, η*_p_*^2^ = 0.25) and for the difference between current and desired QoL (*p* < 0.001, η*_p_*^2^ = 0.23), followed by for mean desired QoL (*p* = 0.34, η*_p_*^2^ = 0.013). These results did not change after correction for age (Wilks’s λ = 0.94, *p* = 0.15) and gender (Wilks’s λ = 0.93, *p* = 0.18).

In summary, depression mainly influences current but not desired QoL in patients with PD. Therefore, the Calman gap (difference between current and desired QoL) is larger in people with depression than in people without depression.

## 4. Discussion

In this study, we applied a new questionnaire to explore the difference between current QoL and desired QoL in relation to different QoL domains. All participants with chronic disorders, as well as controls, reported a gap between current QoL and desired QoL. For patients with PD, the gap was largest with regard to pain and physical functions, such as general health status, physical activities, and fitness.

In general, patients with PD did not seem to have exaggerated expectations regarding QoL. This was demonstrated by the fact that the gap between their current and desired QoL was comparable with that of patients with epilepsy and controls. On average, all patients and controls desired their QoL to be approximately 18% to 22% better. On the other hand, current QoL in patients with PD was poorer and desired QoL was correspondingly lower. However, the adaptation of desired QoL to reality does not take place in the same way for all patients. A difference between desired and current QoL of about 18% was found only in patients with PD who had no depression. Depressed patients with PD, on the other hand, rated their current QoL as significantly worse than did depressed patients with epilepsy and controls, even though desired QoL remained almost unchanged, therefore a larger difference was observed. This gap contributed decisively to the dissatisfaction and poorer QoL of this depressed subgroup. However, because of the cross-sectional design of this study and the lack of objective markers (e.g., Unified PD Rating Scale), we could not determine whether depressed patients had very high expectations or evaluated their current condition very poorly. Patients without depression would have to be monitored longitudinally in order to determine the changes in assessment of current and desired conditions that occur when patients become depressed.

Furthermore, it was interesting to observe which factors determined current and desired QoL. The different models showed that perceived current QoL was essentially determined by the extent of depression. To a lesser extent, however, level of education, living situation, and the presence of a disease (condition) also played a role in the individual assessments of current QoL. In contrast, desired QoL was essentially determined by the presence of a disease (condition), education level, and age. In particular, the presence of PD, lower education level, and higher age was found to be correlated with a lower desired QoL. Depression played no role for desired QoL, which is consistent with the aforementioned phenomenon that depressed patients do not adjust their expectations with regard to current QoL. The strong influence of the presence of a disease on desired QoL can be explained by the fact that one third of the questions asked were about physical functions that are traditionally limited by a disease such as PD. The influence of the professional and educational domains was reflected in the questions about professional position/recognition and lifestyle.

This study had some limitations. Initially, the aim was to quantify the Calman gap; therefore, an explorative design was chosen. Naturally, this limits the validity of confirmatory tests. However, it could be presented as a proof of principle because the questionnaire design revealed interesting aspects of QoL that had previously received little attention. Above all, we demonstrated a clear and valid influence of depression on the Calman gap in patients with PD. Understanding the areas of QoL that are influenced by different factors (such as depression, education) is crucial in order to improve QoL of patients. In addition, because the data were collected anonymously, it was not possible in this study to determine how other cofactors (disease severity, non-motor complaints, cognitive disorders) affect the Calman gap. For the same reason, it was also not possible to detect depression using semi-structured interviews according to the *Diagnostic and Statistical Manual of Mental Disorders*. In interpreting the results, we have to take into account that findings can differ when other measures of depression (e.g., Beck depression inventory) are used. Nevertheless, the PHQ-9 summed-item score method (that we used) at a proposed cut-off point of ≥ 10 has sufficient diagnostic performance for screening purposes [13].

Due to insufficient statistical power, we refrained from a more detailed analysis of the QoL domains that were queried in our questionnaire. For further studies, it would be interesting to know the relevant factors for the physical function, finance, and lifestyle QoL domains. The next steps would therefore be to use the questionnaire (with additional items, if necessary) in better clinically characterized cohorts. In terms of PD, it would be interesting to take relevant cofactors (e.g., distinct non-motor symptoms) into account. Only 63% (*n* = 25/40) of the patients with epilepsy responded to the questionnaire. Thus, our conclusion may not be generalizable to other patients with epilepsy. This exploratory study was not performed to validate (validity, internal reliability) a new questionnaire. Instead, we used an uncommon approach to measure an underreported phenomenon in QoL research. Although we developed our questionnaire from three previously validated questionnaires, the validity or reproducibility of this composite questionnaire needs further studies.

## 5. Conclusions

The study shows that patients with PD do not generally tend to have unrealistically high expectations of QoL. In contrast to PD patients without depression, PD patients with depression had significantly worse QoL than did other subjects, but desired QoL was almost the same, like in the other groups, and hence, they had a larger gap between current and desired QoL. This larger gap in patients with depression contributes decisively to the dissatisfaction and poorer QoL seen in this depressive subgroup.

## Figures and Tables

**Table 1:** Epidemiological and clinical characteristics of the cohort.

Characteristics		All Participants		Controls		Participants with Parkinson’s Disease		Participants with Epilepsy	
Epidemiological Characteristics		Mean	SD	Mean	SD	Mean	SD	Mean	SD
**Age**		64.6	11.8	65.2	10.1	68.3	8.9	50.9	13.7
**PHQ-9 sum score**		8.1	4.9	5.7	4.9	9.6	4.4	7.2	5.1
Clinical characteristics		**n**	**%**	**n**	**%**	**n**	**%**	**n**	**%**
**Gender**	Male	63	44.7	10	25.6	43	55.8	10	40.0
	Female	78	55.3	29	74.4	34	44.2	15	60.0
**Marital status**	Single	8	5.7	1	2.6	2	2.6	5	20.8
	Married	106	75.7	34	87.2	62	80.5	10	41.7
	Partnership	10	7.1	3	7.7	2	2.6	5	20.8
	Divorced, widowed, separated	16	11.4	1	2.6	11	14.3	4	16.7
**Living arrangement**	Alone	19	14.0	3	7.9	12	16.0	4	17.4
	With partner or family	117	86.0	35	92.1	63	84.0	19	82.6
**Occupation**	Employed full-time	16	11.4	8	20.5	3	3.9	5	20.0
	Employed part-time	6	4.3	2	5.1	0	0.0	4	16.0
	Unemployed	6	4.3	2	5.1	4	5.3	0	0.0
	Retired	112	80.0	27	69.2	69	90.8	16	64.0
**Education level**	Low	27	19.1	8	20.5	11	14.3	8	32.0
	Medium	66	46.8	19	48.7	36	46.8	11	44.0
	High	48	34.0	12	30.8	30	39.0	6	24.0
**Depression according to PHQ-9**	Healthy or unremarkable	88	64.7	33	89.1	37	50.0	18	72.0
	Mild	32	23.5	1	2.7	26	35.1	5	20.0
	Moderately severe	13	9.6	2	5.4	10	13.5	1	4.0
	Severe	3	2.2	1	2.7	1	1.4	1	4.0

PHQ-9, Patient Health Questionnaire-9; SD, standard deviation.

**Table 2:** Linear regression analysis: Predictors of mean current quality of life.

	Unstandardized Coefficients		Standardized Coefficients	*t*	*p*
	b	SE	β		
**Entire cohort**					
Constant	77.23	2.19	-	35.16	<0.001
PHQ-9	−1.53	0.23	0.70	−6.73	<0.001
Education level (high)	6.61	2.15	0.15	3.13	0.002
Living situation (alone)	−7.01	2.86	0.09	−2.45	0.016
Condition (Parkinson’s disease)	−3.89	2.13	0.05	−1.82	0.071
**Parkinson’s disease**					
Constant	69.74	3.49	-	19.99	<0.001
PHQ-9	−1.12	0.29	0.54	−3.95	<0.001
Education level (high)	7.77	2.85	0.26	2.73	0.008
Living situation (alone)	−9.11	3.81	0.20	−2.39	0.019

PHQ-9 indicates Patient Health Questionnaire-9.

**Table 3:** Linear regression analysis: Predictors of mean desired quality of life.

	Unstandardized Coefficients		Standardized Coefficients	*t*	*p*
	b	SE	β		
**Entire cohort**					
Constant	98.4	4.25	-	23.12	<0.001
Condition (Parkinson’s disease)	−4.80	1.56	0.39	−3.07	0.003
Education level (high)	4.37	1.57	0.32	2.78	0.006
Age	−0.18	0.07	0.28	−2.63	0.010
**Parkinson’s disease**					
Constant	97.74	8.03	-	12.17	<0.001
Education level (high)	6.48	2.14	0.48	3.03	0.003
Marital status (single, divorced, widowed)	−6.45	2.70	0.30	−2.39	0.019
Age	−0.24	0.12	0.22	−2.01	0.048

**Table 4:** Linear regression analysis: Predictors of the difference between current and desired quality of life.

	Unstandardized Coefficients		Standardized Coefficients	*t*	*p*
	b	SE	β		
**Entire cohort**					
Constant	26.58	4.94	-	5.38	<0.001
PHQ-9	1.45	0.18	0.81	8.10	<0.001
Age	−0.23	0.07	0.12	−3.14	0.002
Gender (male)	−3.96	1.70	0.07	−2.33	0.021
**Parkinson’s disease**					
Constant	10.22	3.10	-	3.31	0.001
PHQ-9	0.88	0.26	0.46	3.46	0.001
Gender (male)	−7.12	2.48	0.32	2.87	0.005
Marital status (single, divorced, widowed)	18.67	7.87	0.22	2.37	0.020

PHQ-9 indicates Patient Health Questionnaire-9.

## Supplementary Materials

The following are available online at https://www.mdpi.com/2077-0383/9/5/1496/s1, Figure S1: A. Current Quality of Life (QoL) in people with Parkinson’s disease for all 15 items (mean and standard deviation). B. Desired QoL in people with Parkinson’s disease for all 15 items (mean and standard deviation); Figure S2: Difference between current and desired Quality of Life (QoL) in people with Parkinson’s disease for all 15 items (mean and standard deviation), Table S1: Quality of Life Questionnaire; Table S2: Values for the 15 QoL questionnaire items for current state, desired state, and the difference between current and desired state.

## Abbreviations

PHQ-9	Patient Health Questionnaire-9
PD	Parkinson’s disease
QoL	Quality of life
