# Supplementary material for: Current and Desired Quality of Life in People with Parkinson’s Disease: The Calman Gap Increases with Depression
Source: J Clin Med. 2020 May 15;9(5):1496. doi: 10.3390/jcm9051496 (PMC7290961; doi:10.3390/jcm9051496)
Supplement: Supplementary file 1 [file jcm-09-01496-s001.pdf]

Supplement

Table S1. Quality of Life Questionnaire.

Topic	Item	100%	0%
**Fitness**	How fit do you feel?	I could run a marathon tomorrow.	I cannot get up without help.
**State of health**	How do you assess your state of health?	Extremely good	Extremely bad
**Pain**	Do you suffer from pain?	Every day	Never
**Climbing stairs**	Do you have difficulties climbing stairs?	I can easily climb as many stairs as I want.	I cannot climb stairs without help.
**Physical activities**	Are you affected by your current state of health during daily activities (kneeling, picking up something, shopping, cleaning up, etc.)?	No, I am not restricted in any way.	Yes, I am completely restricted
**Occupational position**	With the position I have in my profession, I am … (Retired persons please rate former occupation)	Extremely satisfied	Extremely dissatisfied
**Occupational stress**	With the extent of my professional demands and burdens I am … (Retired persons please rate former occupation)	Extremely satisfied	Extremely dissatisfied
**Occupational esteem**	I experience appreciation from my colleagues at work. (Retired persons please rate former occupation)	Every day I experience appreciation	I do not experience esteem on any day
**Income**	With my wage / income I am…	Extremely satisfied	Extremely dissatisfied
**Lifestyle**	With my lifestyle I am…	Extremely satisfied	Extremely dissatisfied
**Financial budget**	Are you satisfied with the financial means that you have at your disposal?	Yes, I can afford anything I want.	No, I cannot afford any luxury
**Workday quitting time**	With the way I spend my evening, I am…	Extremely satisfied	Extremely dissatisfied
**Hobbies**	Do you have time for your hobbies?	Yes, I can do my hobbies whenever I want.	No, I never get to do my hobbies.
**Friends**	How often do you meet with friends, relatives or work colleagues?	Every day	Never
**Partnership**	The quality of my partnership makes me…	Extremely satisfied	Extremely dissatisfied

Table S2. Values for the 15 QoL questionnaire items for current state, desired state and the difference between current and desired state.

Questionnaire item	Subjects with epilepsy		Subjects with Parkinson's disease		Control subjects	
	Mean	SD	Mean	SD	Mean	SD
			**Current QoL**			
Fitness	55.8	23.6	41.6	22.4	63.7	22.2

State of health	60.6	19.8	48.1	20.9	71.7	16.0
Climbing stairs	74.8	22.0	53.2	26.6	80.1	20.7
Physical activities	77.0	23.7	48.2	23.2	77.2	20.7
Pain	58.6	29.8	53.0	31.1	62.2	30.5
Occupational position	73.5	25.7	70.7	26.9	77.4	21.2
Occupational stress	73.4	21.2	58.1	30.3	73.2	23.0
Occupational esteem	70.8	24.0	76.3	24.4	74.9	23.3
Income	49.7	34.9	66.4	21.9	69.1	19.9
Financial budget	62.4	30.5	67.4	23.5	75.8	20.2
Workday quitting time	71.1	21.6	64.9	21.7	70.8	20.7
Hobbies	78.2	19.7	64.9	24.2	63.8	26.6
Friends	57.2	22.7	57.4	21.8	56.4	24.5
Lifestyle	75.0	21.0	63.8	24.3	70.1	20.3
Partnership	75.0	28.7	75.3	23.5	75.4	24.9
**Desired QoL**						
Fitness	77.0	23.6	72.9	20.5	80.3	15.1
State of health	86.6	13.9	80.6	14.7	87.0	11.5
Climbing stairs	93.1	11.6	81.4	17.8	91.0	12.3
Physical activities	91.0	15.2	80.7	18.4	91.0	12.5
Pain	92.1	14.4	85.7	14.8	90.8	13.0
Occupational position	94.7	9.4	87.2	16.4	90.6	9.8
Occupational stress	93.4	10.8	81.4	20.7	89.1	11.9
Occupational esteem	93.6	10.8	86.5	17.5	89.7	11.6
Income	91.8	14.1	87.7	11.5	92.2	9.1
Financial budget	91.4	11.9	87.4	11.2	91.7	8.1
Workday quitting time	92.2	10.1	85.0	10.7	88.7	10.7
Hobbies	93.4	8.1	82.4	16.9	88.3	12.1
Friends	78.4	17.7	75.5	15.6	74.9	16.5
Lifestyle	94.4	6.8	88.0	11.8	88.4	9.9
Partnership	90.0	13.9	88.4	11.9	92.0	8.6
**Difference between current and desired QoL**						
Fitness	21.2	19.3	31.8	19.3	17.2	21.6
State of health	26.0	13.6	32.7	18.9	16.1	14.8
Climbing stairs	19.4	16.7	27.4	19.7	11.4	13.0
Physical activities	14.0	17.1	32.2	20.1	14.4	15.7
Pain	33.8	26.3	32.9	29.4	27.2	27.0
Occupational position	21.2	21.4	17.0	20.0	14.2	18.9
Occupational stress	20.0	16.2	22.7	24.1	16.6	20.0
Occupational esteem	22.8	24.3	9.7	14.5	14.0	19.0
Income	39.5	31.7	21.5	19.5	23.9	19.6
Financial budget	29.0	27.1	20.4	19.4	15.9	16.9
Workday quitting time	21.1	20.0	20.0	18.7	18.7	19.2
Hobbies	15.2	19.8	17.5	21.6	25.4	25.4
Friends	21.2	20.2	18.2	18.3	18.5	23.9
Lifestyle	19.6	17.3	24.8	23.8	18.8	17.2
Partnership	15.5	21.2	13.6	19.3	17.9	23.9

Prell et al.: Current and desired quality of life in people with Parkinson's Disease: the Calman Gap increases with depression

QoL indicates quality of life; SD, standard deviation.

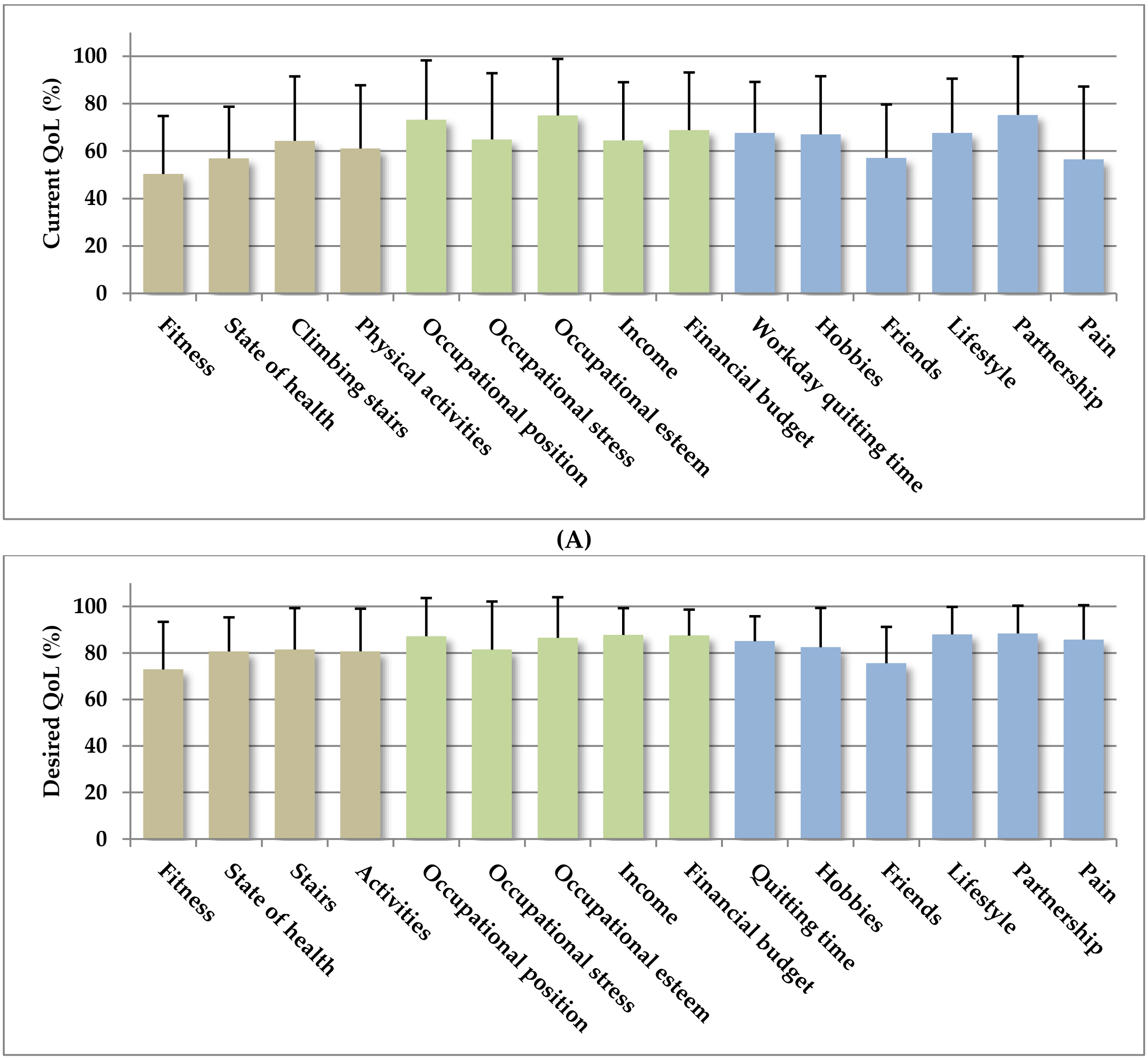

(A)

(B)

Figure S1. A. Current Quality of Life (QoL) in people with Parkinson´s disease for all 15 items (mean and standard deviation). **B.** Desired QoL in people with Parkinson´s disease for all 15 items (mean and standard deviation).

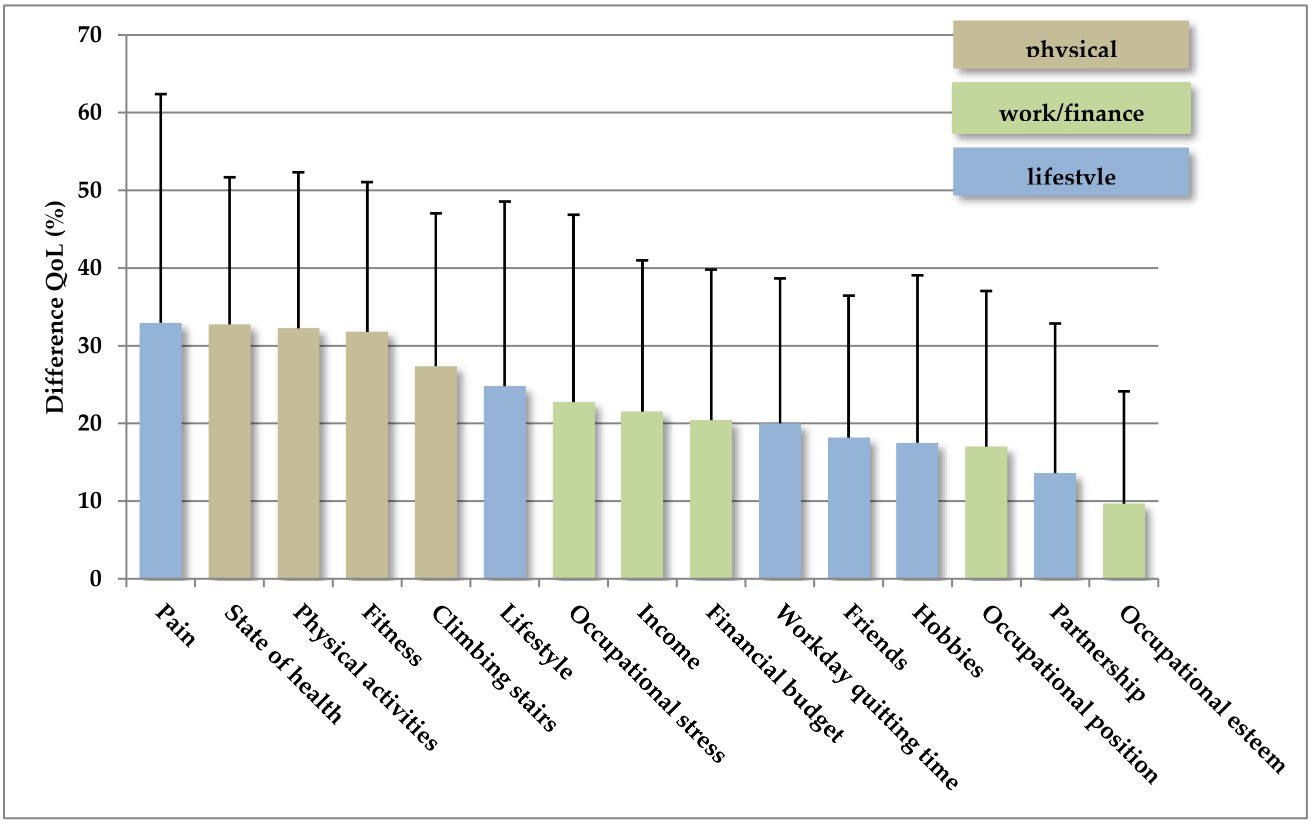

Figure S2. Difference between current and desired Quality of Life (QoL) in people with Parkinson´s disease for all 15 items (mean and standard deviation).
